# Learning about successfully implemented sustainability policies abroad increases support for sustainable domestic policies

**DOI:** 10.1038/s41598-024-62275-w

**Published:** 2024-05-25

**Authors:** Matejas Mackin, Trevor Spelman, Adam Waytz

**Affiliations:** Kellogg School of Management, Northwestern University, Evanston, USA

**Keywords:** Climate-change mitigation, Psychology and behaviour

## Abstract

Anthropogenic climate change poses an existential threat to life on Earth, hastening the need to generate support for sustainability policies. Four preregistered studies (total *N* = 2524) tested whether informing United States citizens about the successful implementation of sustainability policies abroad increased support for similar domestic policies. Studies 1 and 2 found that learning about the successful implementation of sustainability policies (reducing automobile use, using wind energy) abroad increased (1) support for similar domestic policies, (2) intentions to modify behavior to facilitate the adoption of sustainability policies, and (3) behavioral support for sustainability policies. Study 3 found that learning about sustainability policies in both WEIRD (Western, Educated, Industrialized, Rich, Democratic) (France) and non-WEIRD (Colombia) countries increased support for similar domestic policies. Study 4 found that learning about sustainability policies abroad increased support for domestic policy proposals that would impact participants’ city of residence. Overall, these findings suggest that educating citizens about the implementation of sustainability policies abroad can bolster support for domestic policies that combat climate change.

## Introduction

Climate change presents an immediate and escalating threat to human societies and ecosystems worldwide^1,2^. Consequences of climate change are already occurring, with rising sea levels, increased frequency of extreme weather events, and shifting global ecosystems^3,4^. If humans do not act immediately to mitigate the effects of climate change, the consequences will become increasingly dire for life on Earth^5^.

Though many U.S. citizens believe addressing climate change is important^6^, structural factors can inhibit people’s ability to engage in more sustainable behaviors^7–9^. For example, many U.S. towns and cities do not have adequate infrastructure to support sustainable forms of transportation^10^ (e.g., biking, walking), and heavy reliance on fossil fuels for energy presents U.S. citizens with few sustainable means of powering their homes^11^. Addressing climate change requires bolstering public support for structural changes that encourage sustainable behaviors.

Garnering support for large-scale societal structural change is challenging for at least three reasons: (1) people seek to avoid uncertainty^12,13^, (2) people often prefer the status quo^14–17^ (and their current behavioral routines), and (3) people are not always aware of alternative behaviors^18^. Novel, structural changes that require individuals to rely less on fossil fuels involve considerable uncertainty, may alter people’s status quo routines, and may involve solutions that people have not yet considered.

One possible way to overcome these barriers and increase support for structural change is to increase citizens’ awareness of the successful implementation of sustainability policies in other countries. This method may increase support for similar domestic policies by, for example, reducing uncertainty about the policy implementation, educating people about the benefits of such changes, and providing examples of alternative sustainable societal structures they had not previously considered.

The current research presents a novel intervention that can increase support for structural changes to improve sustainability. Specifically, four studies tested and demonstrated that learning about successfully implemented sustainability policies abroad increased support for similar domestic sustainability policies. These studies focused on policies that re-imagine two major societal structures that contribute significantly to climate change: (1) transportation and (2) energy infrastructure. We chose these domains because they contribute substantially to U.S. greenhouse gas emissions.

First, in 2021, transportation accounted for the largest portion (29%) of U.S. greenhouse gas emissions, with 58% of transportation emissions coming from light-duty vehicles^19^ (e.g., passenger cars, trucks). Moreover, road transportation accounts for the largest share of premature deaths linked to emissions, with 53,000 early fatalities annually attributed to the exhaust emitted by cars and trucks^20^. Despite its importance, citizens often find sustainable transportation costly because most U.S. cities prioritize automobile transit^10^. Thus, policies that promote sustainable transportation infrastructure development, such as more bike paths and sidewalks, are crucial for mitigating climate change and saving lives.

Second, the U.S.’s energy infrastructure is highly reliant on fossil fuels, which exacerbate climate change^21^. Though many citizens may wish to power their homes sustainably, it is often not feasible given the limited availability of energy alternatives in the United States. Expanding wind energy infrastructure would substantially reduce air pollution and could save the U.S. up to $500 billion from reduced pollutants and $280 billion in natural gas costs by 2050^22^. Thus, bolstering support for developing wind energy infrastructure in the U.S. is important. Given that extant transportation and energy infrastructure in the U.S. contribute significantly to climate change, we tested whether our intervention encourages support for sustainably restructuring these sectors.

Four preregistered studies (total *N* = 2524) tested whether informing United States citizens about successfully implemented sustainability policies in other countries increased support for similar domestic sustainability policies. Studies 1–4 are methodologically similar in that they all explore how learning about sustainability policies in another country affects attitudes and behaviors towards similar policies domestically. All studies contain slight deviations from one another to conceptually replicate and extend our core finding in different ways: Study 2 examines a different sustainability policy (wind energy instead of sustainable transportation) from the other studies and also differs by including a behavioral donation measure to assess support for the policy. Study 3 contains a different treatment condition from the other studies in which participants learn about sustainability policies in a non-WEIRD^23^ (Western, Educated, Industrial, Rich, Democratic) reference country (Bogota, Columbia). Study 4 differs from the other studies by recruiting residents of Chicago, Illinois, to examine our findings in a context concerning a local sustainability policy that directly affects the study population. By using a broadly consistent methodology with important alterations across studies, these studies build on each other to suggest that educating citizens about the implementation of sustainability policies abroad can bolster support for similar domestic policies.

We preregistered all studies at AsPredicted.Org. All preregistrations, Supplementary materials (including demographic information, exclusion criteria, stimuli, items, and additional analyses), and data can be accessed at: https://researchbox.org/2278 [passcode: THLPJX]. The Institutional Review Board at a Midwestern university approved all experimental protocols, and the researchers carried out all methods per the guidelines and regulations the IRB put forth.

## Study 1

Study 1 tested whether learning about the successful implementation of sustainable urban planning policies that prioritized biker and pedestrian transportation over automobile traffic in Paris, France, increased U.S. citizens’ support for similar policies in the U.S. We predicted that participants who learned about the implementation of these policies abroad would be more likely to support similar policies in the U.S.

### Participants

We recruited 600 Prolific respondents who were U.S. residents. We recruited enough participants to detect a small effect of our intervention (*d* = 0.25) with 80% power. We excluded seven participants for failing a manipulation check (“*What was the topic of the article you read?*”), leaving a final sample size of 593. Among participants in the final sample, 290 were Democrats, 322 were female, 420 were White/Caucasian, and the mean age of participants was thirty-seven. Although we did not target a nationally representative sample, in all studies we sought to recruit a diverse sample across political affiliation, gender, ethnicity, and age. In this study and all subsequent studies, we obtained informed consent from all participants at the outset of the study.

### Procedure

We randomly assigned participants to one of two conditions: learning vs. control. In the learning condition, participants read an article about how Paris implemented urban planning policies that transformed automobile infrastructure to prioritize biker and pedestrian transportation. In the control condition, participants read an article describing contemporary urban planning in the United States, which emphasizes automobile transit. Throughout our studies, we used various control conditions that matched the content of treatment conditions without evoking ideas about the successful implementation of policies in other countries. The online supplement provides all experimental stimuli used, as well as exclusion criteria and demographic information for each study.

### Measures

After reading the article, participants completed four measures that assessed support for domestic sustainable urban planning policies.

#### Attitude measure

We measured participants’ attitudes towards proposed changes in domestic urban planning using a three-item measure, which asked participants, “How strongly do you support the following measures: (1) Re-purposing highways for pedestrians and cyclists? (2) Re-purposing on-street car parking for bicycle lanes? (3) Making biking safer and more accessible in American cities?” We measured all items on a seven-point Likert scale (1 = *Not at all,* 7 = *Strongly support*; α = .86).

#### Behavioral intentions

We measured participants’ willingness to engage in sustainable behaviors using a three-item measure that asked participants, “How willing would you be to… (1) Decrease your reliance on automobile transportation in the future? (3) Vote in favor of initiatives that promote the walkability and bikeability of cities and towns in your state?” We measured all items on a seven-point Likert scale (1 = *Not at all,* 7 = *Extremely willing*; α = .91).

#### Urban planning priorities

We measured the extent to which participants wanted the United States to prioritize urban planning initiatives that support biker and pedestrian versus automobile transportation using a single item, which asked, “Over the next 50 years, to what extent do you think that United States infrastructure plans should prioritize…” (1 = *Biker, pedestrian, and public transportation*, 7 = *Automobile transportation*).

#### Behavioral measure

We included a single-item behavioral measure that asked participants whether they would rather read an article about sustainable or automobile transit at the end of the study.

### Results

**Figure 1:**
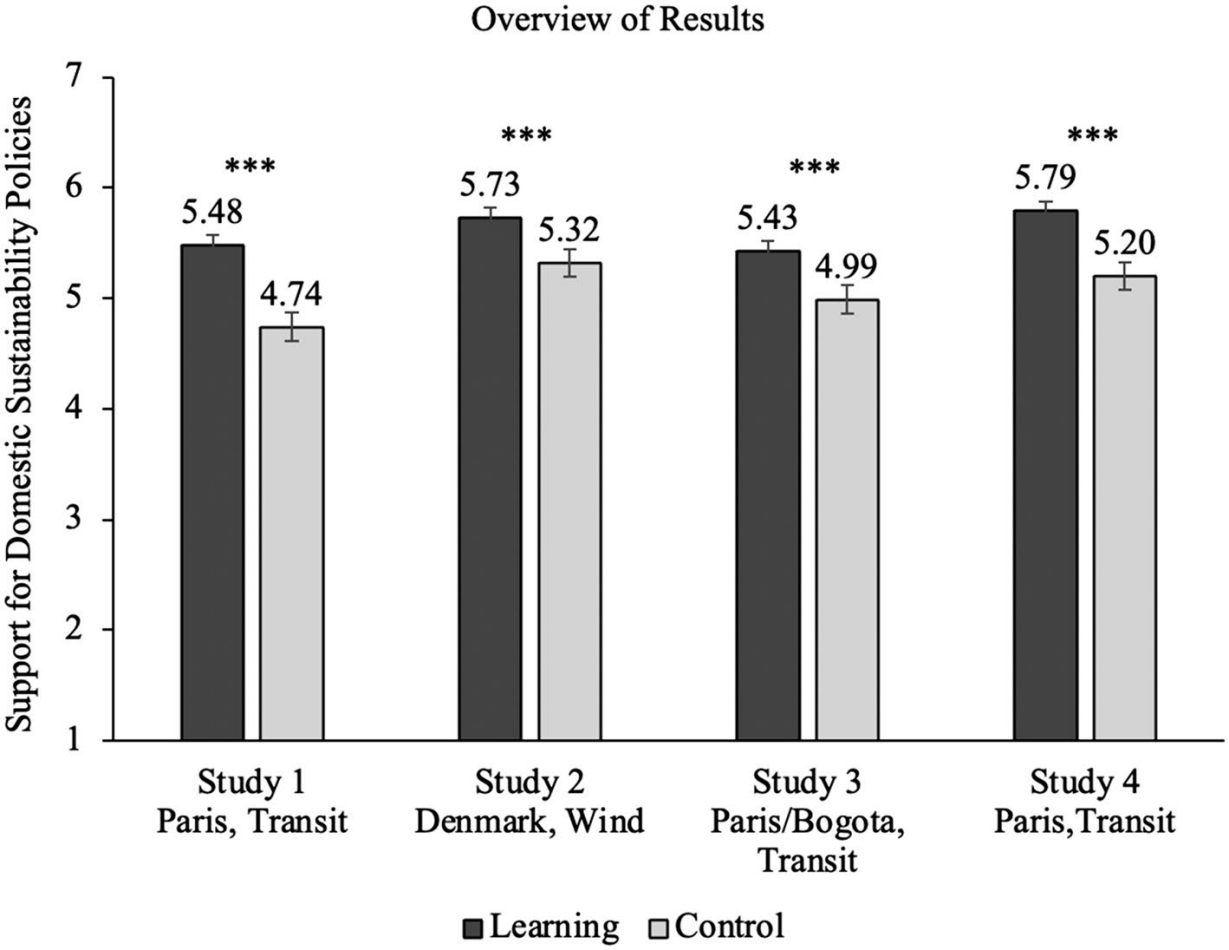
Comparison of support for domestic sustainability policies between learning and control conditions across all studies.

Participants in the learning condition supported domestic sustainable urban planning policies more than participants in the control condition, *t*(591) = 6.12, *p* < .001, *d* = 0.50, expressed more willingness to adopt sustainable transportation behaviors, *t*(591) = 2.97, *p* = .003, *d* = 0.24, and were more likely to say that the U.S. should prioritize biker, pedestrian, and public transportation compared to participants in the control condition, *t*(591) = 4.11, *p* < .001, *d* = 0.34. Finally, participants in the learning condition were more likely to choose to read an article about sustainable urban planning policies in the United States (85%) than participants in the control condition (74%), *χ*^*2*^ (1, 593) = 10.60, *p* < .001. We present the means and standard deviations for all items across studies in Table 1. The results for our key dependent measure—support for domestic sustainability policies—by condition across all studies is shown in Fig. 1.

**Table 1:** Means and standard deviations for items across all studies.

Study	*N*	Condition	Item	Mean (SD)
1	593	Learning	Sustainability policy support	5.48 (1.41)
		Control	Sustainability policy support	4.74 (1.56)
		Learning	Behavioral intentions	5.24 (1.60)
		Control	Behavioral intentions	4.84 (1.72)
		Learning	Priority*	3.07 (1.51)
		Control	Priority*	3.61 (1.69)
2	596	Learning	Sustainability policy support	5.73 (1.47)
		Control	Sustainability policy support	5.32 (1.69)
		Learning	Behavioral intentions	5.08 (1.62)
		Control	Behavioral intentions	4.74 (1.77)
		Learning	Allocation	81.3 (29.7)
		Control	Allocation	75.4 (33.0)
3	885	Learning—Paris	Sustainability policy support	5.43 (1.48)
		Learning—Bogota	Sustainability policy support	5.42 (1.41)
		Control	Sustainability policy support	4.99 (1.51)
		Learning—Paris	Behavioral intentions	5.11 (1.74)
		Learning—Bogota	Behavioral intentions	5.14 (1.60)
		Control	Behavioral intentions	4.78 (1.72)
		Learning—Paris	Priority*	2.99 (1.52)
		Learning—Bogota	Priority*	3.10 (1.59)
		Control	Priority*	3.51 (1.67)
		Learning—Paris	Allocation	60.2 (30.8)
		Learning—Bogota	Allocation	60.2 (29.6)
		Control	Allocation	56.2 (30.9)
4	450	Learning	Sustainability policy support	5.79 (1.39)
		Control	Sustainability policy support	5.20 (1.46)

*Lower scores indicate higher priority.

### Discussion

Overall, participants who learned about the successful implementation of sustainable urban planning policies abroad expressed more support for similar domestic policies, more willingness to adopt sustainable transportation habits, and more interest in learning about sustainable urban planning.

## Study 2

Study 2 conceptually replicated Study 1 using a different policy domain in a different reference location in the learning condition. Specifically, Study 2 examined whether learning about the successful implementation of wind energy infrastructure in Denmark increased support for policies that promote wind energy infrastructure in the U.S.

### Participants

We recruited 600 Prolific respondents who were U.S. residents. We recruited enough participants to detect a small effect of our intervention (*d* = 0.25) with 80% power. We excluded four participants for failing a manipulation check, leaving a final sample size of 596. Of these, 301 were Democrats, 307 were female, 409 were White/Caucasian, and the mean age of participants was thirty-eight. We obtained informed consent from all participants at the outset of the study.

### Procedure

We randomly assigned participants to one of two conditions: a learning condition or a control condition. In the learning condition, participants read an article describing wind energy infrastructure in Denmark. In the control condition, participants read an article about existing energy infrastructure in the United States.

### Measures

After reading the article, participants completed three measures that assessed support for domestic sustainable energy policies.

#### Attitude measure

We measured participants’ attitudes towards proposed changes in domestic energy infrastructure policies using a three-item measure (α = .92), which asked participants, “How strongly do you support the following measures: (1) Reducing reliance on fossil fuels in the U.S.? (2) Building more wind turbines to increase the amount of wind energy generated in the U.S.? (3) Prioritizing wind energy infrastructure over fossil fuel energy infrastructure in the U.S.?” Participants responded to all items on a seven-point Likert scale (1 = *Not at all,* 7 = *Strongly support*).

#### Behavioral intentions

We measured participants’ willingness to engage in sustainable behaviors and behaviors that would promote sustainable structural changes to energy infrastructure in the United States using a five-item measure (α = .92), which asked participants, “How willing would you be to… (1) Sign a petition showing that you support the development of wind energy infrastructure in the United States? (2) Sign up for a program that uses wind energy to power your own home? (3) Vote in favor of policies that increase U.S. reliance on wind energy? (4) Financially invest in wind energy projects or companies? (5) Vote in favor of policies that decrease U.S. reliance on fossil fuels?” All items were measured on a seven-point Likert-scale (1 = *Not at all,* 7 = *Extremely willing).*

#### Behavioral measure

We collected a single-item behavioral measure. We measured how our manipulation affected participants’ behaviors by giving participants a $1.00 bonus to allocate between an organization that promotes the development of wind energy infrastructure in the U.S. and an organization that promotes responsible fossil fuel use in the U.S.

### Results

Compared to participants in the control condition, participants in the learning condition were more supportive of sustainable domestic energy infrastructure development, *t*(594) = 3.19, *p*  = .002, *d* = 0.26, expressed more willingness to adopt sustainable energy consumption behaviors, *t*(594) = 2.41, *p* = .021, *d* = 0.20, preferred that the United States prioritize wind energy over fossil fuel development more strongly, *t*(594) = 3.41, *p* < .001, *d* = 0.28, and allocated more money to the domestic wind energy organization, *t*(594) = 2.29, *p* = .022, *d* = 0.19. See Table 1 for all means and standard deviations.

### Discussion

Overall, Study 2 further demonstrated that learning about successfully implemented sustainability policies abroad increases support for similar domestic policies. Study 2 extended the findings of Study 1 by using another reference country (Denmark) in another domain critical to combatting climate change (energy infrastructure and consumption).

## Study 3

Studies 1 and 2 showed that participants support domestic sustainability policies more after learning about sustainability policies in a WEIRD (Western, Educated, Industrial, Rich, Democratic) reference country (i.e., France, Denmark) compared to the control condition. Study 3 tested whether learning about the successful implementation of sustainable urban planning policies in a non-WEIRD reference country (Bogota, Colombia) increased support for sustainable urban planning policies in the U.S. similarly to learning about urban planning in a WEIRD reference country (Paris, France; as in Study 1).

### Participants

We recruited 899 Prolific respondents who were U.S. residents. We recruited enough participants to detect a small effect of our intervention (*d* = 0.25) with 80% power. We excluded fourteen participants for failing a manipulation check, leaving a final sample size of 885. Of these, 411 were Democrats, 453 were female, 673 were White/Caucasian, and the mean age of participants was forty-two. Participants gave informed consent at the outset of the study.

### Procedure

We randomly assigned participants to one of three conditions: a WEIRD learning condition, a non-WEIRD learning condition, or a control condition. In the WEIRD learning condition, participants read the same article describing the successful implementation of sustainable urban planning policies in Paris, France that was used in Study 1. In the non-WEIRD learning condition, participants read an article describing the successful implementation of sustainable urban planning policies in Bogota, Colombia. In the control condition, participants read the same control article that was used in Study 1.

After reading the article, participants completed four measures that assessed support for domestic sustainable urban planning policies. These included the same three-item attitudinal measure (α = .86), three-item behavioroid measure (α = .91), and single-item priority measure from Study 1, and a single-item resource allocation task in which participants allocated a $1 bonus between an organization promoting biking in U.S. cities (People for Bikes) and an organization supporting safe and efficient automobile transit in America (Transportation for America).

### Results

A main effect of condition emerged on support for sustainable domestic urban planning policies, *F*(2, 882) = 8.56, *p* < .001, *η*^*2*^ = 0.02, intent to adopt sustainable transportation behaviors, *F*(2, 882) = 4.16, *p* = .016, *η*^*2*^ = 0.01, and support for prioritizing biking and walking over automobile transit, *F*(2, 882) = 8.56, *p* < .001, *η*^*2*^ = 0.02. Replicating Study 1, participants who learned about urban planning in Paris supported sustainable domestic urban planning policies more than participants in the control condition, *t*(586) = 3.53, *p* < .001, *d* = 0.29, were more willing to adjust their behavior, *t*(586) = 2.33, *p* = .020, *d* = 0.19, and showed greater support for prioritizing biking and walking, *t*(586) = 3.91, *p* < .001, *d* = 0.32.

Further, participants who learned about urban planning in Bogota were also more supportive of sustainable domestic urban planning policies than participants in the control condition, *t*(586) = 3.60, *p* < .001, *d* = 0.30, were more willing to adjust their behavior, *t*(586) = 2.64, *p* = .008, *d* = 0.22, and showed greater support for prioritizing biking and walking, *t*(586) = 3.06, *p* = .002, *d* = 0.25. Participants who learned about urban planning in Paris and Bogota similarly supported sustainable domestic urban planning policies, *t*(592) = 0.04, *p* = .970, *d* < 0.01, were similarly willing to adjust their behavior, *t*(592) = 0.21, *p* = .832, *d* = 0.02, and similarly prioritized biking and walking, *t*(601) = 0.82, *p* = .414, *d* = 0.07. We did not find a main effect of condition on the amount of money that participants allocated to a charity promoting biking and walking (People for Bikes), *F*(2, 882) = 1.63, *p* = .197, *η*^*2*^ = 0.01. We present the means and standard deviations for all items in Table 1.

### Discussion

Overall, participants who learned about the successful implementation of sustainable urban planning policies in either a WEIRD (Paris) or non-WEIRD (Bogota) country supported domestic sustainability policies to a similar extent and similarly expressed willingness to adjust their behavior to accommodate these policies more than participants who learned about mainstream urban planning in the United States. The lack of significant difference between the WEIRD and non-WEIRD conditions provides initial evidence to suggest that learning about the successful implementation of sustainability policies in both WEIRD and non-WEIRD reference countries can encourage support for domestic sustainability policies.

## Study 4

Studies 1, 2, and 3 demonstrated that learning about the successful implementation of sustainability policies abroad increased support for similar policies in the U.S. However, it is unclear whether this type of learning increases support only for policies that affect the United States in general or for sustainability policies that affect one’s day-to-day life (in one’s own city). Study 4 thus examined whether our intervention impacted support for local sustainability policies in participants’ own city of residence. Specifically, we tested whether telling residents of Chicago, Illinois, about successfully implemented sustainability policies in Paris, France, increased their support for similar sustainability policies in Chicago.

### Participants

We recruited 544 respondents from a university-run participant pool in which all participants currently lived in Chicago, Illinois. Given the limited size of this pool, we aimed to recruit the maximal number of participants possible. We recruited enough participants to detect a small effect of our intervention (*d* = 0.30) with 80% power (after making all exclusions). We excluded fifty-seven participants who failed an attention check at the outset of the survey, twenty-four duplicate responses, and twelve participants who failed a comprehension check following the manipulation, yielding a final sample size of 450 participants. Of these, 314 were Democrats, 269 were female, 309 were White/Caucasian, and the mean age of participants was forty. We obtained informed consent from all participants at the outset of the study.

### Procedure

We randomly assigned participants to either a learning condition or a control condition. In the learning condition, participants read the same article describing the successful implementation of sustainable urban planning policies in Paris, France, as in Study 1. In the control condition, participants read an article that described how Paris became a global center for fashion. Whereas Studies 1, 2, and 3 used a control condition that described the status quo in the United States, the control condition in this study held several key elements of the treatment condition constant. Specifically, the description provided to participants in both the learning and the control conditions in Study 4 included the same reference location (Paris) as well as an innovation to the status quo—but only the treatment condition described an innovation in sustainability policy.

### Measures

After reading the article, participants completed one focal measure of support for domestic sustainable urban planning policies. Additionally, participants completed two exploratory measures that assessed behavioroid (five items) and behavioral (two items) support for sustainable urban planning policies in Chicago. We discuss exploratory measures in the Supplementary Materials.

#### Attitude measure

We measured participants support for sustainable urban planning policies in Chicago using a three-item measure (α = .80), which asked participants, “How strongly do you support the following policies… (1) Re-designing Lake Shore Drive to make it more accessible to pedestrians and bikers, (2) Creating new public transit options along Lake Shore Drive, (3) Reducing the number of cars on Lake Shore Drive to create a safer and more pleasant experience for those walking and biking by the lake?” [Lake Shore Drive is a major highway that runs through the city of Chicago, Illinois]. We measured all items on a seven-point Likert scale (1 = *Not at all;* 7 = *Strongly support*).

### Results

Participants in the learning condition were more supportive of domestic sustainable urban planning policies than participants in the control condition (*t*(448) = 4.35, *p* < .001, *d* = 0.41). We did not find significant differences between conditions for any of our exploratory measures (See Supplementary Materials for all analyses).

### Discussion

Overall, participants who learned about the successful implementation of sustainable urban planning policies abroad were more supportive of similar domestic policies in their own city compared to participants in the control condition who learned about innovation abroad. The pattern of results for other measures were in the same direction of our main effect between conditions but did not reach statistical significance.

## Discussion

The current work introduced a novel intervention that increased support for sustainability policies in the U.S. Four preregistered studies found evidence that learning about the successful implementation of sustainability policies abroad increased attitudinal and behavioral support for domestic sustainability policies. Moreover, we found promising evidence in support of the potential generalizability of this effect. Specifically, we found that this effect persisted across policy domains (urban planning, wind energy), with WEIRD and non-WEIRD reference countries (France, Denmark, Colombia), and when individuals considered actual sustainability policies proposed in their own city. Our work adds to a growing body of literature that uses psychological interventions to combat the climate crisis^24,25^.

We note that our intervention appears to work for both liberals and conservatives (see Supplementary Materials for analyses with political ideology). Thus, our intervention may be a promising avenue to increase support for sustainability policies among less climate-interested groups, such as U.S. conservatives.

Although we observed consistent effects on self-reported support for sustainability policies, our intervention clearly impacts some behaviors (e.g., desire to learn more about sustainability policies, allocating more resources to sustainability organizations) more than others. However, given that the effects were in the hypothesized direction for all measures across all studies, future studies with larger samples may reveal more consistent effects of our intervention on various behavioral measures. Future research can also better assess any disconnects between support for sustainable policies and behaviors.

Another direction for future research is to examine the effect in other populations. Although we suspect our intervention will affect non-U.S. samples similarly to U.S. samples, future research can test its generalizability more explicitly. For instance, feelings of nationalism may moderate the efficacy of our intervention, making it less effective in highly nationalistic countries where other countries’ success is less relevant to the success of one’s home country. Our intervention may also be particularly effective in countries that value multiculturalism and are highly open to alternative policy innovations that originate in places outside one’s home country.

Finally, although we demonstrate an immediate shift in attitudes and intentions, future research can test the extent to which these effects persist over time. Existing work has shown that similar brief messaging interventions that encourage sustainable attitudes and behaviors have durable effects on energy consumption that can last weeks after implementation^26^, suggesting the possibility that an intervention like ours may endure as well, at least to an extent.

This intervention holds the potential to advance support for sustainability policies. Politicians and governments aiming to implement such policies stand to benefit by informing citizens about analogous successful initiatives in other countries, underscoring the tangible benefits accrued by those nations. This approach not only provides policymakers with a persuasive tool to garner public backing but also fosters a sense of feasibility and desirability among citizens. Moreover, learning about successful sustainability initiatives elsewhere has the potential to shape individual behavior. When citizens learn about the successful implementation of sustainability policies in other nations, there may be a behavioral shift at the grassroots level. Individuals, inspired by these success stories, may be more inclined to support similar domestic sustainability policies and adopt environmentally friendly practices in their daily lives.

### Supplementary Information

Supplementary Information.

## Data Availability

All data used in analyses and the R code for analyses performed by the authors can be accessed at the following link: https://researchbox.org/2278. Passcode: THLPJX.
