# Supplementary material for: Learning about successfully implemented sustainability policies abroad increases support for sustainable domestic policies
Source: Sci Rep. 2024 May 25;14:11983. doi: 10.1038/s41598-024-62275-w (PMC11128015; doi:10.1038/s41598-024-62275-w)
Supplement: Supplementary file 1 — Supplementary Information. [file 41598_2024_62275_MOESM1_ESM.docx]

**Supplementary Materials**

Table of Contents

[STUDY 1 2](#_Toc151572793)

[**Descriptive information** 2](#_Toc151572794)

[**Exclusion criteria** 2](#_Toc151572795)

[**Learning Article** 2](#_Toc151572796)

[**Control Article** 2](#_Toc151572797)

[**Dependent Measures** 3](#_Toc151572798)

[STUDY 2 4](#_Toc151572799)

[**Descriptive information** 4](#_Toc151572800)

[**Exclusion criteria** 4](#_Toc151572801)

[**Learning article** 4](#_Toc151572802)

[**Control article** 4](#_Toc151572803)

[**Dependent measures** 5](#_Toc151572804)

[STUDY 3 7](#_Toc151572805)

[**Demographic information** 7](#_Toc151572806)

[**Exclusion criteria** 7](#_Toc151572807)

[**WEIRD learning article** 7](#_Toc151572808)

[**Non-WEIRD article** 7](#_Toc151572809)

[**Control article** 8](#_Toc151572810)

[**Dependent measures** 8](#_Toc151572811)

[STUDY 4 10](#_Toc151572812)

[**Descriptive information** 10](#_Toc151572813)

[**Exclusion criteria** 10](#_Toc151572814)

[**Learning article** 10](#_Toc151572815)

[**Control article** 11](#_Toc151572816)

[**Dependent measures** 11](#_Toc151572817)

[**Additional Analyses** 12](#_Toc151572818)

[FURTHER ANALYSES 14](#_Toc151572819)

[**Political ideology (Study 1)** 14](#_Toc151572820)

[**Political ideology (Study 2)** 14](#_Toc151572821)

[**Political ideology (Study 3)** 15](#_Toc151572822)

[**Political ideology (Study 4)** 15](#_Toc151572823)

#

# **STUDY 1**

## **Descriptive information**

| Variable | Control | Learning |
| --- | --- | --- |
| Sample size | 303 | 290 |
| Gender | 45% female | 44% female |
| Mean age | 38 | 37 |
| Race | 75% White/Caucasian | 67% White/Caucasian |
| Political Ideology | 48% Democrat | 50% Democrat |

## **Exclusion criteria**

We recruited 602 participants from Prolific. 9 participants were excluded for failing a manipulation check after reading the article: “What was the topic of the article you read?” Of these, 1 was in the control condition, and 8 were in the learning condition.

## **Learning Article**

[This article was adapted from: https://www.youtube.com/watch?v=GSQSBoHmG8s&t=1s&ab_channel=Distilled]

In the 1990s, Paris was one of the most polluted, traffic-clogged cities in the world. Today, the city is a world leader in sustainable urban development. Here's the story of how Paris took back its streets from cars.

One of the first efforts to reduce car dependency in Paris was the launch of a bike-share service called Velib. These bike-share services are in cities around the world today. But when Paris launched the program in 2007 it was revolutionary.

When the city launched Velib it had a problem: there was very little bike infrastructure. That made it dangerous to ride, which in turn made biking unattractive to most people. All of this changed in 2015 when the city invested 150 million euros in new cycling infrastructure. The following year, the city closed a highway along the Seine river and turned it into a pedestrian area and bike path. A few years later, Paris converted the Rue de Rivoli -- one of the busiest roads in the city -- into a bike highway. Paris has also replaced much of the city's car parking with bike parking and pedestrian areas. In 2020, Paris announced that the city would remove a staggering 72% of its on-street car parking to accommodate cyclists.

These changes made a huge difference. Between 2020 and 2021, cycling traffic in Paris grew by 70%. Car ownership has fallen from 60% to 35% in the last two decades. All of this has helped Paris cut its carbon emissions by about 20% over that same period.

## **Control Article**

Automobiles have become a common mode of transportation on American streets. They are used by millions of people every day to travel to different destinations. Automobiles come in various sizes and shapes, ranging from small compact cars to large trucks and SUVs. They are powered by internal combustion engines, which use gasoline or diesel fuel to propel the vehicle forward.

Automobiles are subject to certain rules and regulations on American streets. Drivers must abide by speed limits and traffic signals to ensure safety for themselves and others. Automobiles must also be registered and insured to operate on public roads.

To maintain the road network, the government invests a significant amount of resources in building and maintaining infrastructure like highways, bridges, and interchanges. This infrastructure enables automobiles to travel longer distances more efficiently and at higher speeds, making it possible for people to access different parts of the country.

In conclusion, automobiles are a ubiquitous feature of American streets, used by millions of people every day to travel to different destinations.

## **Dependent Measures**

1. Attitudinal measures: **How strongly do you support the following policies?** (*1 = Not at all, 7 = Strongly support*)
   1. Re-purposing highways for pedestrians and cyclists
   2. Re-purposing on-street car parking for bicycle lanes
   3. Making biking safer and more accessible in American cities
2. Behavioroid measures: **How willing would you be to…** (*1 = Not at all, 7 = Extremely willing*)
   1. Decrease your reliance on automobile transportation in the future?
   2. Increase your reliance on walking, biking, and public transportation in your day-to-day life?
   3. Vote in favor of initiatives that promote the walkability and bikeability of cities and towns in your state?
3. Priority measure
   1. **Over the next 50 years, to what extent do you think that United States infrastructure plans should prioritize…** (*1 = Biker, pedestrian, and public transportation, 7 = Automobile transportation*).
4. Behavioral measure
   1. Later in the study, you may be asked to read an article and then we will ask you some questions about that article. We will allow you to choose which article you will be asked to read. The titles are listed below. **Which of the following articles do you want to read in the next part of the study?** (*1 = Re-imagining the American commute: The future of biker, pedestrian, and public transit, 2 = From horse-and-buggy to horsepower: How the automobile revolutionized American transportation*).

# **STUDY 2**

## **Descriptive information**

| Variable | Control | Learning |
| --- | --- | --- |
| Sample size | 300 | 296 |
| Gender | 48% female | 46% female |
| Mean age | 38 | 38 |
| Race | 71% White/Caucasian | 66% White/Caucasian |
| Political Ideology | 49% Democrat | 52% Democrat |

## **Exclusion criteria**

We recruited 600 participants from Prolific. 4 participants were excluded for failing a manipulation check after reading the article: “What was the topic of the article you read?” Of these, 1 was in the control condition, and 3 were in the learning condition.

## **Learning article**

In the 1970s, Denmark, like many countries, relied heavily on fossil fuels and faced environmental challenges. However, the country embarked on a remarkable journey towards sustainable energy. Investing in wind energy became a central focus.

Denmark capitalized on wind power potential. As Denmark recognized the need for change, the nation shifted its approach and prioritized developing renewable energy sources. They started building wind farms onshore and offshore, harnessing the power of the wind to generate clean electricity.

This shift towards wind energy brought significant benefits to Denmark. The country reduced its reliance on fossil fuels, thereby reducing greenhouse gas emissions and combating climate change. Denmark's commitment to wind energy also resulted in a substantial decrease in carbon emissions from transportation. Wind energy provides significant amounts of electricity for electric vehicles, making them more accessible and affordable. As a consequence, Danish people emit three times less CO2 from their cars compared to Americans. Moreover, wind energy played a vital role in achieving energy independence and bolstering the country's renewable energy sector.

## **Control article**

The United States, like many other countries, has long relied on fossil fuels as a crucial component of its energy infrastructure. The United States possesses abundant reserves of coal, oil, and natural gas, which have played a significant role in meeting the nation's energy demands. These fossil fuels have been instrumental in powering industries, transportation systems, and electricity generation across the nation.

The extensive use of these energy sources has helped fuel economic growth, create job opportunities, and contribute to energy security. The availability of domestic fossil fuel reserves has provided the United States with considerable energy independence, leading to reduced dependence on foreign energy imports.

Furthermore, the existing infrastructure for extracting, refining, and distributing fossil fuels has provided a reliable and established energy framework. While the United States is increasingly diversifying its energy mix and exploring renewable alternatives, fossil fuels have historically provided a stable and cost-effective energy solution for the country.

## **Dependent measures**

1. Attitudinal measures: **How strongly do you support the following policies?** (*1 = Not at all, 7 = Strongly support*)
   1. Reducing reliance on fossil fuels in the US
   2. Building more wind turbines to increase the amount of wind energy generated in the US
   3. Prioritizing wind energy infrastructure over fossil fuel energy infrastructure in the US
2. Behavioroid measures: **How willing would you be to…** (*1 = Not at all, 7 = Extremely willing*)
   1. Sign a petition showing that you support the development of wind energy infrastructure in the United States?
   2. Sign up for a program that uses wind energy to power your own home?
   3. Vote in favor of policies that increase US reliance on wind energy?
   4. Financially invest in wind energy projects or companies?
   5. Vote in favor of policies thar decrease US reliance on fossil fuels?
3. Behavioral measure (0 cent to 100 cent scale)
   1. We’d like you to allocate $1 between two charitable organizations described below. It is up to you to decide how much of the $1 each organization gets. Once the study is complete, we will randomly select 20 participants’ donation allocations and make donations to these organizations on their behalf. Charity 1: Wind Energy Foundation (WEF): WEF is a nonprofit organization focused on educating policymakers and the public about the benefits of wind energy. They collaborate with stakeholders to promote the integration of wind power into the U.S. electric grid and advocate for policies that encourage wind energy expansion. Charity 2: American Petroleum Institute (API): API is the largest national trade association representing the oil and gas industry in the United States. They advocate for policies that support responsible development of oil and natural gas resources. API promotes industry standards, conducts research, and engages in public outreach. **How would you like to allocate the $1 between the two organizations? Use the slider scale below to indicate, in cents, how much of the $1 you’d like each organization to receive.**

# **STUDY 3**

## **Demographic information**

| Variable | Control | WEIRD Learning | Non-WEIRD Learning |
| --- | --- | --- | --- |
| Sample size | 291 | 297 | 297 |
| Gender | 50% female | 50% female | 54% female |
| Mean age | 42 | 41 | 42 |
| Race | 77% White/Caucasian | 75% White/Caucasian | 76% White/Caucasian |
| Political Ideology | 45% Democrat | 48% Democrat | 47% Democrat |

## **Exclusion criteria**

We recruited 899 participants from Prolific. 14 participants were excluded for failing a manipulation check after reading the article: “What was the topic of the article you read?” Of these, 5 were in the control condition, 3 were in the WEIRD learning condition, and 6 were in the non-WEIRD learning condition.

## **WEIRD learning article**

In the 1990s, Paris was one of the most polluted, traffic-clogged cities in the world. Today, the city is a world leader in sustainable urban development. Here's the story of how Paris took back its streets from cars.

One of the first efforts to reduce car dependency in Paris was the launch of a bike-share service called Velib. These bike-share services are in cities around the world today. But when Paris launched the program in 2007 it was revolutionary.

When the city launched Velib it had a problem: there was very little bike infrastructure. That made it dangerous to ride, which in turn made biking unattractive to most people. All of this changed in 2015 when the city invested 150 million euros in new cycling infrastructure. The following year, the city closed a highway along the Seine river and turned it into a pedestrian area and bike path. A few years later, Paris converted the Rue de Rivoli -- one of the busiest roads in the city -- into a bike highway. Paris has also replaced much of the city's car parking with bike parking and pedestrian areas. In 2020, Paris announced that the city would remove a staggering 72% of its on-street car parking to accommodate cyclists.

These changes made a huge difference. Between 2020 and 2021, cycling traffic in Paris grew by 70%. Car ownership has fallen from 60% to 35% in the last two decades. All of this has helped Paris cut its carbon emissions by about 20% over that same period.

## **Non-WEIRD article**

In the 1990s, Bogota, Colombia was one of the most polluted, traffic-clogged cities in the world. Today, the city is a world leader in sustainable urban development. Here's the story of how Bogota took back its streets from cars.

The transformation of Bogotá started with a simple idea: to reclaim the streets from cars. A crucial step was the creation of over 590 kilometers of bicycle paths and pedestrian walkways as viable alternatives for daily travel. Sundays witnessed a remarkable change as more than 120 kilometers of roads closed for seven hours during Ciclovia. This weekly event allowed 1.5 million people of diverse backgrounds to cycle, skate, or walk, while parks turned into hubs for activities like yoga and music.

In 2000, Mayor Enrique Peñalosa introduced the concept of car-free days, an initiative that gained public support and has since become an annual event. This move led to decreased emissions and a reduction in traffic-related fatalities. During these designated days, approximately 600,000 cars stayed off the roads, contributing to improved air quality and reduced congestion.

These changes made a huge difference. In the last 10 years, cycling has increased by 50%. Because of these urban planning developments, the average citizen saves 200 hours in transportation time and more than 10% of their income each year. All of this has helped Bogota cut its carbon emissions by about 28% over that same period.

## **Control article**

Automobiles have revolutionized travel and become a common mode of transportation on American streets. They are used by millions of people every day to travel to different destinations.

Automobiles come in various sizes and shapes, ranging from small compact cars to large trucks and SUVs. Automobiles originally replaced the horse-and-buggy as the primary mode of intra-city travel. They are powered by internal combustion engines, which propel the vehicle forward.

Automobiles are subject to certain rules and regulations on American streets. Drivers must abide by speed limits and traffic signals to ensure safety for themselves and others. Automobiles must also be registered and insured to operate on public roads.

To maintain the road network, the government invests a significant amount of resources in building and maintaining infrastructure like highways, bridges, and interchanges. This infrastructure enables automobiles to travel longer distances more efficiently and at higher speeds, making it possible for people to access different parts of the country.

In conclusion, automobiles are a ubiquitous feature of American streets, used by millions of people every day to travel to different destinations.

## **Dependent measures**

1. Attitudinal measures: **How strongly do you support the following policies?** (*1 = Not at all, 7 = Strongly support*)
   1. Re-purposing highways for pedestrians and cyclists
   2. Re-purposing on-street car parking for bicycle lanes
   3. Making biking safer and more accessible in American cities
2. Behavioroid measures: **How willing would you be to…** (*1 = Not at all, 7 = Extremely willing*)
   1. Decrease your reliance on automobile transportation in the future?
   2. Increase your reliance on walking, biking, and public transportation in your day-to-day life?
   3. Vote in favor of initiatives that promote the walkability and bikeability of cities and towns in your state?
3. Priority
   1. Over the next 50 years, to what extent do you think that United States should invest in infrastructure plans that prioritize… (*1 = Biker, pedestrian, and public transportation, 7 = Automobile transportation*)
4. Behavioral measure (0 cent to 100 cent scale)
   1. We'd like you to allocate $1 between two charitable organizations described below. It is up to you to decide how much of the $1 each organization gets.
      Once the study is complete, we will randomly select 20 participants’ donation allocations and make donations to these organizations on their behalf.
      **Charity 1: People For Bikes (PFB)**: PFB is a nonprofit organization whose mission is to make bike riding safer and better for everyone. PFB seeks to ensure that bikes are prioritized and positioned as a real solution to improve Americans’ health, connect communities, boost local and state economies, strengthen our nation and protect our planet.
      **Charity 2: Transportation For America (TFP)**: TFP is a nonprofit organization whose mission is to improve automobile infrastructure to get people where they need to go safely and efficiently. They advocate for policies that support the maintenance of our current highway infrastructure, prioritizing safety over speed in cities and town, and connecting people to jobs and services through an effective roadway network. 
      How would you like to allocate the $1 between the two organizations? Use the slider scale below to indicate, in cents, how much of the $1 you'd like each organization to receive.

# **STUDY 4**

## **Descriptive information**

| Variable | Control | Learning |
| --- | --- | --- |
| Sample size | 223 | 227 |
| Gender | 62% female | 58% female |
| Mean age | 39 | 40 |
| Race | 69% White/Caucasian | 69% White/Caucasian |
| Political Ideology | 73% Democrat | 67% Democrat |

## **Exclusion criteria**

We recruited 544 respondents from a university-run participant pool in which all participants currently lived in Chicago, Illinois. Exclusions were made in the following order:

- 35 duplicate responses were deleted. That is, some participants tried to take the survey more than once. In cases in which participants took the survey several times, all additional responses after their first response were deleted. Their first response was retained and included in analyses.
- 45 failed the following attention check at the outset of the survey and were not randomized to condition:
  - In order to facilitate our research, we are interested in knowing certain factors about you. We want to know about your reading comprehension on basic instructions. Specifically, we are interested in whether you actually take the time to read the directions; if not, then the data we collect based on your responses will be invalid. So, in order to demonstrate that you have read the instructions, please ignore the next question, and simply write “I read the instructions” in the box labeled "Any comments?" Thank you very much.
- 13 participants were excluded for failing an attention check after reading their randomly assigned article (“What was the topic of the article you read?”)
- 2 participants did not complete the survey.

## **Learning article**

In the 1990s, Paris was one of the most polluted, traffic-clogged cities in the world. Today, the city is a world leader in sustainable urban development. Here's the story of how Paris took back its streets from cars.

One of the first efforts to reduce car dependency in Paris was the launch of a bike-share service called Velib. These bike-share services are in cities around the world today. But when Paris launched the program in 2007 it was revolutionary.

When the city launched Velib it had a problem: there was very little bike infrastructure. That made it dangerous to ride, which in turn made biking unattractive to most people. All of this changed in 2015 when the city invested 150 million euros in new cycling infrastructure. The following year, the city closed a highway along the Seine river and turned it into a pedestrian area and bike path. A few years later, Paris converted the Rue de Rivoli -- one of the busiest roads in the city -- into a bike highway. Paris has also replaced much of the city's car parking with bike parking and pedestrian areas. In 2020, Paris announced that the city would remove a staggering 72% of its on-street car parking to accommodate cyclists.

These changes made a huge difference. Between 2020 and 2021, cycling traffic in Paris grew by 70%. Car ownership has fallen from 60% to 35% in the last two decades. All of this has helped Paris cut its carbon emissions by about 20% over that same period.

## **Control article**

In the early 20th century, Paris was just one of many centers for global fashion, competing with cities like London and Milan. Today, Paris stands unrivaled as the fashion capital of the world. Here's the story of how Paris became the epicenter of global fashion.

The turning point for Paris came when it became known for its incredibly detailed, custom-made outfits in the early 1900s. This followed the founding of Haute Couture – an organization that was central in setting the standards for high-quality fashion. However, in the middle of the 20th century, Paris faced a new challenge. People started to prefer clothes that were ready to buy off the rack instead of custom-made pieces. To keep up with this trend, Paris introduced its own fashion events dedicated to these more accessible styles.

As such, the city launched its first Prêt-à-Porter, or Ready-to-Wear, Fashion Week in 1973. This move diversified Paris' fashion offerings and opened up a new market. Around the same time, the city invested heavily in fashion education. Schools like ESMOD expanded their curricula, attracting students from around the world.

These changes had a significant impact. Today, Paris Fashion Week generates over 1.2 billion euros in annual revenue, and the city is home to over 5,000 fashion showrooms. The percentage of global luxury fashion brands headquartered in Paris has risen from 25% in the early 1990s to over 40% today. All of this has solidified Paris' standing as not just a center of fashion but as the global epicenter of style and design.

## **Dependent measures**

1. Support for biking and pedestrian transportation measures: **How strongly do you support the following policies?** (*1 = Not at all, 7 = Strongly support*)
   1. Re-designing Lake Shore Drive to make it more accessible to pedestrians and bikers
   2. Creating new public transit options along Lake Shore Drive
   3. Reducing the number of cars on Lake Shore Drive to create a safer and more pleasant experience for those walking and biking by the lake
2. Support for discouraging driving measures: **How strongly do you support the following policies?** (*1 = Not at all, 7 = Strongly support*)
   1. Making parking in Chicago more expensive
   2. Lowering speed limits city-wide
   3. Adding more speed bumps throughout Chicago streets
3. Behavioroid measures: **How willing would you be to…** (*1 = Not at all, 7 = Extremely willing*)
   1. Decrease your reliance on automobile transportation in the future?
   2. Increase your reliance on walking, biking, and public transportation in your day-to-day life?
   3. Vote in favor of initiatives that promote the walkability and bikeability of Chicago?
   4. Volunteer with an organization to promote increased biker and pedestrian transportation in Chicago?
   5. Sign a petition showing that you support the re-purposing of Chicago’s Lake Shore Drive for biker and pedestrian use?
4. Priority measure
   1. **Over the next 50 years, to what extent do you think that United States infrastructure plans should prioritize…** (*1 = Biker, pedestrian, and public transportation, 7 = Automobile transportation*).
5. Petition measure
   1. We’re collecting anonymous signatures for a petition of people who support the proposal to re-purpose portions of Chicago’s Lake Shore Drive to prioritize bicycle, pedestrian, and public transit traffic. **Do you want to have your signature anonymously included in this petition?** (1 = *Yes,* 0 = *No*).
6. Information measure
   1. **Would you like more information about organizations in the Chicagoland area that are working to promote bicycle and pedestrian safety and infrastructure?** If you select ‘yes’ we will share relevant links at the end of the study. (1 = *Yes*, 0 = *No*).
7. Donation measure
   1. **Please use the slider scale below to indicate the amount (in cents) that you would like to donate to the Active Transportation Alliance (ATA)** (0 = *0 cents*, 100 = *100 cents*).

## **Additional Analyses**

We preregistered several additional analyses that further explored the scope of our intervention.

1. *Support for discouraging driving measures.* Participants in the implementation and control conditions did not differ significantly in their support for policies that discourage driving in Chicago (*t*(448) = .686, *p* = .49).
2. *Behavioroid measures.* Participants in the implementation and control conditions were similarly willing to adopt sustainable transportation behaviors (*t*(448) = 1.55, *p* = .12).
3. *Priority measure.* Participants in the implementation and control conditions similarly indicated the United States should prioritize biker, pedestrian, and public transportation (*t*(448) = 1.45, *p* = .15).
4. *Petition measure*. 71% of participants in the treatment condition indicated they wanted to sign a petition supporting redefine LSD, vs. 64% in the control condition (*χ^2^* (1, 450) = 1.79, *p* = .18)
5. *Information measure*. 47% of participants in the treatment condition indicated they wanted to read more about progressive urban planning at the end of the survey, vs. 42% in the control condition (*χ^2^* (1, 450) = .766, *p* = .38)
6. *Donation measure.* Participants in the treatment condition (*M* = 64.5, *SD* = 43.3) donated similarly to ATA as participants in the control (*M* = 61.8, *SD* = 44.2) (*t*(446) = .647, *p* = .52)

# **FURTHER ANALYSES**

In this section, we examine whether any of our results are moderated by political ideology. We measured political ideology with a single item:

“Generally speaking, do you think of yourself as a Republican, and Democrat, an Independent, or something else?” (*1 = Republican, 2 = Democrat, 3 = Independent, 4 = Other (please specify)*).

For simplicity, in the following analyses we only analyze differences between Republicans and Democrats. We exclude participants who selected ‘independent’ or ‘other’ from analyses in this section.

Overall, we find that our intervention works similarly for both liberals and conservatives.

## **Political ideology (Study 1)**

1. Attitudinal measure
   1. We do not observe a statistically significant interaction between condition and political ideology on support for sustainability policies (*F*(1, 390) = 1.92, *p* = .17).
2. Behavioroid measure
   1. We do not observe a statistically significant interaction between condition and political ideology on willingness to adjust behaviors to support the adoption of sustainability policies (*F*(1, 390) = .241, *p* = .62).
3. Priority
   1. We do not observe a statistically significant interaction between condition and political ideology on desire to prioritize biker, pedestrian, and public transportation in the future (*F*(1, 390) > .000, *p* = .99).

## **Political ideology (Study 2)**

1. Attitudinal measure
   1. We observe a statistically significant interaction between condition and political ideology on support for sustainability policies (*F*(1, 399) = 5.66, *p* = .02). For conservatives, a t-test revealed that those who learned about wind energy in Denmark were more supportive of domestic sustainability policies (*M* = 4.19, *SD* = 1.77) than those in the control condition (*M* = 3.35, *SD* = 1.69) (*t*(96) = 2.40, *p* = .02, *d* = .48). For liberals, a t-test found no effect of our intervention on support for sustainability policies (*t*(299) = 1.54, *p* = .13).
2. Behavioroid measure
   1. We do not observe a statistically significant interaction between condition and political ideology on willingness to adjust behaviors to support the adoption of sustainability policies (*F*(1, 399) = 1.90, *p* = .17).
3. Allocation
   1. We observe a statistically significant interaction between condition and political ideology on amount allocated to a wind energy charity (*F*(1, 399) = 13.0, *p* < .001). For conservatives, a t-test revealed that those who learned about wind energy in Denmark allocated more money to a wind energy charity (*M* = 57.6, *SD* = 38.4) than those in the control condition (*M* = 36.3, *SD* = 29.1) (*t*(96) = 3.10, *p* = .003, *d* = .63). For liberals, a t-test found no effect of our intervention on support for sustainability policies (*t*(299) = .061, *p* = .95).

## **Political ideology (Study 3)**

1. Attitudinal measure
   1. We do not observe a statistically significant interaction between condition and political ideology on support for sustainability policies (*F*(1, 596) = 1.22, *p* = .30).
2. Behavioroid measure
   1. We do not observe a statistically significant interaction between condition and political ideology on willingness to adjust behaviors to support the adoption of sustainability policies (*F*(1, 596) = 1.20, *p* = .30).
3. Priority
   1. We do not observe a statistically significant interaction between condition and political ideology on desire to prioritize biker, pedestrian, and public transportation in the future (*F*(1, 596) = .954, *p* = .39).

## **Political ideology (Study 4)**

1. Attitudinal measure
   1. We do not observe a statistically significant interaction between condition and political ideology on support for sustainability policies (*F*(1, 334) = .302, *p* = .58).
